# Assessing the Weed-Suppressing Potential of Cotton Chromosome Substitution Lines Using the Stair-Step Assay

**DOI:** 10.3390/plants10112450

**Published:** 2021-11-13

**Authors:** Mary Gracen Fuller, Sukumar Saha, David M. Stelly, Johnie N. Jenkins, Te Ming Tseng

**Affiliations:** 1 Department of Plant and Soil Science, Mississippi State University, Starkville, MS 39762, USA; maf572@msstate.edu; 2 Genetics and Sustainable Agriculture Research Unit, USDA-ARS, Starkville, MS 39762, USA; sukumar.saha@usda.gov (S.S.); johnie.jenkins@usda.gov (J.N.J.); 3 Department of Soil and Crop Sciences, Texas A&M University, College Station, TX 77843, USA; stelly@tamu.edu

**Keywords:** allelopathy, *Gossypium hirsutum*, chromosome substitution, sustainable weed management

## Abstract

Palmer amaranth is a problematic common weed species, especially in cotton. With the wide use of chemical herbicide and herbicide-tolerant transgenic cotton lines, Palmer amaranth populations have developed tolerance to commonly used herbicides. It is imperative to develop alternative weed control methods to slow the evolution of herbicide-resistant weed populations and provide new strategies for weed management. Eleven chromosome substitution (CS) cotton lines (CS-B26Lo, CS-T17, CS-B16-15, CS-B17-11, CS-B12, CS-T05sh, CS-T26Lo, CS-T11sh, CS-M11sh, CS-B22sh, and CS-B22Lo) were screened for weed-suppressing abilities in this study. The cotton lines were tested using the established stair-step assay. Height (cm) and chlorophyll concentration (cci) were measured for each plant in the system. The most significant variation in Palmer amaranth height reduction among the CS lines was observed 21 days after establishment. CS-B22sh (76.82%) and CS-T26Lo (68.32%) were most effective in reducing Palmer amaranth height. The cluster analysis revealed that CS-B22sh, and CS-T26Lo were clustered in one group, suggesting similar genetic potential with reference to Palmer amaranth growth and development. CS-B22sh showed novel genetic potential to control the growth and development of Palmer amaranth, a problematic weed in cotton fields. Future experimentation should implement more parameters and chemical testing to explore allelopathic interactions among CS lines and Palmer amaranth.

## 1. Introduction

Weed Science is a discipline focused on plants that are considered a nuisance [1]. The definition of what is regarded as a weed largely depends on the human perspective and is generally considered a plant growing somewhere it is not desired [2,3]. Today, one of the most troublesome weeds facing farmers and weed scientists is Palmer amaranth (*Amaranthus palmeri*), a broad-leaved, herbaceous dicot capable of prolific seed production and aggressive early seasonal growth [4,5]. Competition imposed by weeds affects the crop directly and indirectly by (a) reducing fiber quality, (b) reducing crop yield, (c) increasing production costs, (d) reducing irrigation efficiency, and (e) serving as hosts and habitats for pests such as insects, rodents, nematodes, and disease-causing pathogens. Palmer amaranth negatively affects many economically important crops, including soybean, sorghum, and cotton [5]. Furthermore, Palmer amaranth is considered the most problematic weed species in eight cotton-producing states, including Mississippi [6,7], and has been observed to reduce cotton lint up to 54% [5]. Palmer amaranth also decreases harvesting proficiency to 2.4% when the weed is present at a density of 3260 weeds ha^−1^ [8].

Palmer amaranth and other weeds found in cotton fields have been traditionally managed through appropriate cultivation, pre-planting, and timed herbicide applications (preemergence and postemergence herbicide application) [6,8], the last of which has become more prevalent in modern agricultural practice due to technological advancements [9]. The compound glyphosate is a key ingredient in Bayer’s Roundup Ready herbicide, a common and effective tool in managing weeds in many economically important agricultural fields such as cotton and soybean [10]. First introduced to the market in 1974, the herbicide effectively interrupts the Shikimate Pathway by downregulating the 5-enolpyruvylshikimate-3-phosphate synthase (EPSPS) enzyme. Elucidated by Srinivasan et al. (1956), the Shikimate pathway is crucial for plants to produce aromatic amino acids such as tyrosine and phenylalanine, in which an interruption results in plant death [11,12].

Glyphosate revolutionized agricultural weed management due to the initial belief that the herbicide was not toxic in mammals and relatively inactive in soil [3]. The use of glyphosate has exponentially increased in conjunction with the development and introduction of glyphosate-resistant (GR) crops in the 1990s, including cotton, soybean, and rice [1,12]. It has been found that glyphosate has adverse side effects not previously considered. For example, many herbicides using glyphosate often mix phytotoxic chemicals with additives such as surfactants, which directly impact the effectiveness of the herbicide mixture [13]. Research conducted by Mesnage, Bernay, and Séralini (2013) suggested that phytotoxic chemicals mixed with adjuvants are 10,000 times more toxic to mammalian mitochondrial activity than phytotoxic chemicals alone [14].

Furthermore, in many agricultural systems, glyphosate is used exclusively for weed control [6]. The overuse of and reliance on glyphosate and other herbicides without rotation in many agricultural systems [3,6,15,16] has led to the development of herbicide resistance in over 200 weedy biotypes all over the world, including the problematic Palmer amaranth [17]. Glyphosate-resistant Palmer amaranth has primarily been managed using glufosinate, dicamba, and 2,4-D herbicides [18], in conjunction with 2,4-D and dicamba-resistant cotton varieties [19]. Due to the rapid evolution of resistant Palmer amaranth, it is reasonable to assume that Palmer amaranth populations will become resistant to glufosinate, dicamba, and 2,4-D herbicides, as was elucidated with glyphosate [19]. To curb the evolution of herbicide-resistant (HR) weed populations, alternative weed control techniques must be developed to supplement chemical weed management in agriculture [20].

Numerous studies on rice (*Oryza sativa*), wheat (*Triticum aestivum*), sunflower (*Helianthus annuus*), and canola (*Brassica napus*) crop varieties have illustrated the capability of some plants to possess weed-suppressing abilities, also known as allelopathy [21,22]. Allelopathy is an observed phenomenon in which secondary metabolites, produced by biochemical pathways of one plant type, inhibit the growth of neighboring plants, thereby reducing competition and increasing success, growth, and fecundity of the source plant [21,23]. This phenomenon can be utilized in conjunction with chemical control techniques to effectively suppress weed competition in agricultural fields and lessen the pressure on chemical control management techniques. Allelopathic crop varieties are up-and-coming for the modern frontier of agriculture because the chemicals produced by the plant of interest are generally biodegradable and safer than conventional herbicides [23]. Although no research has been conducted on the allelopathic effect of cotton on weed species, studies have indicated the ability of cotton varieties to produce allelochemicals. In 1996, Hoffman et al. studied cotton that was observed to inhibit the growth of weeds, including Palmer amaranth [24,25]. Additionally, research conducted by Gui-Ying et al. (2015) detected four phenolic acid compounds in extracts of cotton roots from fields that had been monocropped for an extended period (>10 years); these phenolic acids were classified as p-hydroxybenzoic acid, ferulic acid, gallic acid, and vanillin [25]. Phenolic compounds such as sorgoleone from sorghum have been demonstrated to reduce barnyardgrass and velvetleaf growth by up to 50% [24]. Total phenolic acid from sunflower (at 600 µg/g soil) resulted in up to 68% inhibition of redroot pigweed germination [26]. Due to the suppression of weed growth, when in the presence of phenolic allelochemicals produced by crops of interest, it is reasonable to assume that cotton varieties capable of producing phenolic compounds may possess weed-suppressive potential. To cultivate this characteristic in cotton plants, it is imperative to identify cotton lines that can produce high levels of allelochemicals. They can then be entered into selective breeding programs to develop cotton varieties with substantial weed suppressive ability. Weed-suppressing cotton varieties can contribute to sustainable cotton production and restrain the evolution of HR weeds.

The cotton genome, while large (2n = 52) [27], is mainly composed of homogenous gene segments [28]. The long-term domestication and refinement of *Gossypium hirsutum* cultivars are suspected to be among the key causes for their marked decline in intraspecific polymorphism [29]. The resulting genetic uniformity constrains the effectiveness of efforts by growers, agronomists, and geneticists to address novel problems, such as HR weed populations. Numerous chromosome substitution (CS) cotton lines have been collaboratively developed and tested; each is characterized by the interspecific introgression of unique sets of alleles from alternative cotton species [27]. Initially engineered to improve fiber quality in Upland cotton (*Gossypium hirsutum*), the CS lines were created by substituting a portion of the chromosome with the mirroring chromosome portion present in wild or exotic species of cotton (*Gossypium barbadense, G. mustelinum, G. darwinii, G. tomentosum*). Each CS line contains about 4% of the donor species’ genome, with the remaining portion being largely isogenic to the TM-1 parent [30,31]. However, a few CS lines, e.g., CS-B16-15, were derived from tertiary monosomic recurrent backcross parents that were *G. hirsutum*, but not necessarily isogenic to TM-1, so the respective CS lines would not necessarily be isogenic to TM-1 or other CS lines. The lines have been observed to maintain epistatic genetic effects, altering the expression of traits in unprecedented ways [32].

In preliminary greenhouse experiments (unpublished), the 50 CS lines were screened for their competitive ability. The competitive lines were determined to be: CS-B26Lo, CS-T17, CS-B16-15, CS-B17-11, CS-B12, CS-T05sh, CS-T26Lo, CS-T11sh, CS-M11sh, CS-B22sh, and CS-B22Lo. These lines were further phenotyped in this study. The main objective of the project was to phenotype competitive CS lines in a greenhouse using the stair-step structure to observe potential weed-suppressing characteristics. It is expected for more competitive lines to reduce Palmer amaranth height and chlorophyll concentrations significantly more than non-competitive lines.

## 2. Results

Weed suppressive or otherwise competitive chromosome substitution (CS) cotton lines were determined using the reduction in mean height reduction and chlorophyll concentration of Palmer amaranth, the receiver plant species. The entire system was run for a total of 21 days after establishment (DAE). Height reduction was observed to be statistically different 14 DAE. For this reason, height reduction values were measured starting at 14 DAE. Palmer amaranth leaves were too small to measure chlorophyll concentration without irreparable damage to the plant at 1, 7, and 14 DAE. For this reason, chlorophyll concentration was measured at 21 DAE.

### 2.1. Height Reduction

**Figure 1:**
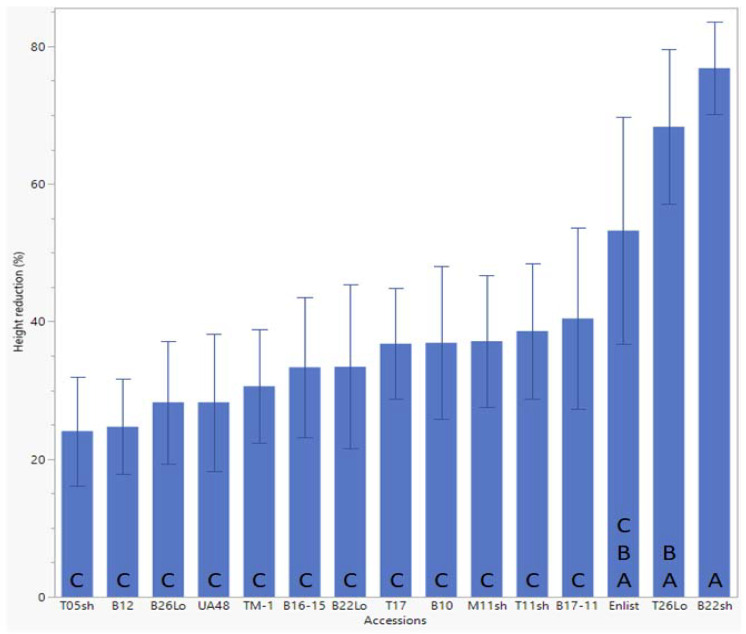
Palmer amaranth height reduction by cotton chromosome substitution (CS) lines at 21 days after establishment (DAE). Data were analyzed using JMP software, where *p* was calculated to be 0.0336. A. B. C. stands for the result of comparison of means by a student’s t-test. The results showed that CS-B22sh performed better than most of the CS lines tested and the control lines UA48 and TM-1.

Height-reduction values for both runs were pooled and analyzed at each day of data collection (DOE, 7, 14, and 21 DAE) using ANOVA, where *p* < 0.05 (Table 1). There were no significant differences in Palmer amaranth height reduction 7 or 14 DAE. On 21 DAE, significant differences in Palmer amaranth height reduction among the CS lines (T26Lo and B22sh) and UA48 (Figure 1). A *p*-value of 0.0336 was calculated. The top-performing line was determined to be CS-B22sh, which reduced Palmer amaranth height by nearly 77%. This line reduced Palmer amaranth height approximately 69% more than the lowest-performing line, CS-T05sh, with an overall Palmer amaranth height reduction value of 24.06%. Additionally, CS-B22sh reduced Palmer amaranth height reduction nearly 30.1% more than the Enlist^®^ cotton (~53% Palmer amaranth height reduction), approximately 60% more than TM-1 (~31% Palmer amaranth height reduction), and ~63% more than UA48 (~28% Palmer amaranth height reduction). The CS line CS-T26Lo also performed exceptionally well in terms of Palmer amaranth height reduction with a mean value of more than 68%.

### 2.2. Chlorophyll Reduction

Chlorophyll reduction values for both runs at 21 DAE were pooled and analyzed using ANOVA. Analysis indicated no significant differences among CS lines, UA48, TM-1, and Enlist^®^. Although not statistically significant, Palmer amaranth chlorophyll reduction was 38% and 55% greater than UA48 and TM-1, respectively.

### 2.3. Cotton Reduction

On 14 DAE, analyses of variance revealed significant differences among CS line height reduction due to weedy interactions. Comparison of means revealed CS-B22Lo height was reduced more by Palmer amaranth than all other CS lines, where *p* = 0.0358. Furthermore, on 21 DAE, CS-B22Lo suffered increased height reduction compared with the lowest-performing CS line with a probability value of 0.0304. There were no significant data for cotton height reduction on the day of establishment. Furthermore, there were no significant differences in cotton chlorophyll reduction between the CS lines, UA48, Enlist^®^, and TM-1 (Table 2).

### 2.4. Hierarchical Clustering and Principal Component Analysis

**Figure 2:**
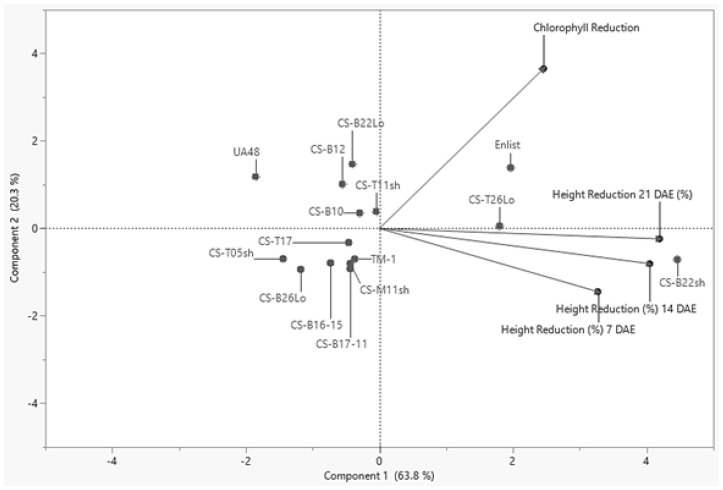
Principal Component Analysis (PCA) with five components: (1) Palmer amaranth mean height reduction 7 days after establishment (DAE) (%), (2) Palmer amaranth mean height reduction 14 DAE (%), (3) Palmer amaranth mean height reduction 21 DAE (%), (4) Palmer amaranth mean chlorophyll reduction 21 DAE (%), (5) mean susceptibility (%). Three groups were formed, guided by K-clustering analysis. The first group is composed of lines: CS-B22Lo, CS-B12, CS-B10, CS-T11sh, and UA48. This group maintained median Palmer amaranth chlorophyll reduction values. The second group was composed of CS-T05sh, CS-B26Lo, CS-B16-15, CS-B17-11, CS-M11sh, CS-T17, and TM-1. This group did not have high reduction values for Palmer amaranth chlorophyll concentration or height reduction of any days of data collection. The third group was composed of CS-T26Lo, CS-B22sh, and Enlist^®^. This group maintained high Palmer amaranth height reduction values.

Clustering analyses were performed utilizing the following five parameters (Table 1): Palmer amaranth mean height reduction 7 DAE (%), Palmer amaranth mean height reduction 14 DAE (%), Palmer amaranth mean height reduction 21 DAE (%), and Palmer amaranth chlorophyll concentration reduction 21 DAE (%). The parameters were pooled and analyzed using a hierarchical cluster guided by K-clustering analysis. Three separate groups emerged. Principal Component Analysis (PCA) guided by hierarchical clustering indicates that 63.8% of the variation in the weed-suppressing characteristics can be attributed to component 1, and 20.3% can be attributed to component 2 (Figure 2). The first group was composed of lines: CS-B22Lo, CS-B12, CS-B10, CS-T11sh, and UA48. This group maintained median Palmer amaranth chlorophyll reduction values ranging from approximately 21% to 16.5%. The second group was composed of CS-T05SH, CS-B26Lo, CS-B16-15, CS-B17-11, CS-M11sh, CS-T17, and TM-1. This group did not have high reduction values for Palmer amaranth chlorophyll concentration or height reduction of any days of data collection. The third group was composed of CS-T26Lo, CS-B22sh, and Enlist^®^. This group represents cotton accessions with high Palmer amaranth height reduction values.

## 3. Discussion

A current weed science challenge is developing new biotechnologies and crops that can combat herbicide-resistant weed populations while maintaining low mammalian toxicity and low residual time in soil. Competitive or allelopathic cotton varieties have the potential to serve as a powerful tool for farmers. The cotton CS lines were tested using the established stair-step structure methodology, which provided a scope to study the effect of individual CS lines on the growth and development of Palmer amaranth weed without any interference of other external factors in the greenhouse. The top-performing CS lines were determined to be CS-B22sh and CS-T26Lo. Enlist was also determined to be grouped with the two competitive CS-lines in further analyses.

*Gossypium tomentosum*, or Hawaiian cotton, is in the primary gene pool for cotton species and closely related to Upland cotton (*G. hirsutum*); however, Hawaiian cotton exhibits marked differences in terms of markers, isozymes, and phenotype. The species was reported by Brubaker et al. (1999) to produce thin, coarse fiber that is generally brown and strong [33]. Furthermore, Hawaiian cotton is incredibly heat-resistant, likely because this species evolved in the Hawaiian Islands, characterized by dry, rocky coastal habitats [30]. Our results indicated that CS-T26Lo is one of the two top-performing lines for reducing the height of Palmer amaranth. It is possible that Hawaiian Cotton biotypes developed the cryptic alleles for weed suppressing ability to compete with other plant species under conditions in which there was low water and nutrient availability. Due to the intense selection pressure on fiber traits placed on Hawaiian Cotton lines during the domestication process, many beneficial traits have been lost in Upland cotton. This line of reasoning suggests that CS-T26Lo will be a useful tool for targeted introgression of lost traits in Upland cotton improvement.

In 2018 Awasthi and a group of researchers investigated potential epistatic effects of CS-B lines by crossing six CS-B lines, including CS-B22sh, in a half diallel. Fiber analysis showed that CS-B22sh maintained significantly higher micronaire values than the TM-1. In mature fibers, lower micronaire values are more desirable. Furthermore, it was determined that CS-B22sh had an average fiber strength (kN m kg^−1^) of 2.0. It was predicted that crosses of CS-B lines with CS-B22sh would result in negative additive fiber strength effects. Most CS-B22sh hybrids maintained lower fiber uniformity ratios than other hybrids, further supporting additive gene action that decreases fiber quality associated with the short arm of chromosome 22 in Hawaiian cotton [34]. In this study, B22sh was significantly more competitive than most of the CS lines screened. Resources for growth and reproduction are not infinite within a plant. In concordance with plant resource allocation theory, fewer resources will be devoted to growth as a plant dedicates more energy and resources to competition [35]. While CS-B22sh was one of the most effective lines in Palmer amaranth height reduction, this CS line also was one of the CS lines which exhibited significant height reduction in the experiment. CS-B22sh mean height was reduced by 14.41%. Additionally, CS-T26Lo was one of the top-performing lines in terms of Palmer amaranth height reduction but was also significantly reduced 14 days after establishment with a reduction value of 26.93% (Table 2). Chromosome substitution lines exhibiting more competitive or allelopathic characteristics may dedicate more resources to defense mechanisms than reproduction or growth, the latter of which is suggested by this study’s results.

The results indicate CS-B22sh and CS-T26Lo were among the top two lines, even compared to the herbicide-tolerant transgenic cotton Enlist^®^, in terms of Palmer amaranth height. The cluster analysis revealed that Enlist^®^ cotton, CS-B22sh, and CS-T26Lo were clustered in one group, suggesting similar genetic potential in reducing the growth and development of Palmer amaranth. It could be implied that genes associated with CS-B22sh and CS-T26Lo are more sensitive to competition with weeds and likely produce potential allelochemicals to inhibit weed growth and development. The discovery of cotton lines with the genetic potential to impede weed growth and development will provide economic benefit to the farmers. Allelopathy is closely linked to stressors within the environment [36]. For this reason, it is believed that allelopathic test results are highly dependent on the conditions in which the plants are tested. The stair-step method in our experiment provided an ideal opportunity to detect the effects of individual CS lines on the growth and development of Palmer amaranth in a controlled greenhouse condition without any interference from other external factors.

The stair-step structure outlined by Schumaker et al. (2020) calls for the use of sand to grow the plant in a pot to avoid negligible cation exchange capacity effects from other chemicals for change in phenotype on the plants in experiments [37,38]. While a nominal cation exchange capacity is a valuable soil characteristic when assessing the potential of allelopathic chemicals leaching from the potted plants through the connected tube to affect the effect, it is not conducive for proper cotton nutrient regiments. Additionally, cotton is a sensitive plant and displays nutrient deficiency symptoms on soils sufficient for other crops [39]. We avoided this nutrient deficiency for the plants in the stair-step system by providing Hogland solution at certain time intervals to all pots to prevent nutrient deficiency in plants. Any observed greenhouse phenomenon may be altered in a field setting due to many external factors in field experiments justified by our stair-step method to study the effect of the CS lines on Palmer amaranth growth and development.

While allelopathic cultivars have not yet been achieved in agronomic cotton varieties, allelopathic interactions have been observed to be successful when using cover crops. For example, cover crop success in rye (*Secale cereale*), wheat (*Triticum aestivum*), sorghum (*Sorghum bicolor*), and barley (*Hordeum vulgare*) is suggested to be a result of allelopathic soil residues [40]. In 1983, Putnam and associates grew rye, wheat, sorghum, and barley up to 50 cm. The crops were then removed through herbicide applications or freezing. Following cover removal, residues were left in the fields, resulting in 95% weed control for up to 60 days following cover crop removal [41]. Furthermore, rye has been observed to produce hydroxamic acid, a well-documented allelochemical, from roots and shoots under biotic stress. This chemical has been observed to reduce competition around the plant, thereby facilitating the growth and regeneration of the crop [36].

## 4. Materials and Methods

### 4.1. Materials

This project utilized eleven chromosome substitution (CS) lines that had been created using homologous chromosomes or chromosome arm of *Gossypium baebadense* (CS-B), *Gossypium tomentosum* (CS-T), and *Gossypium mustellinum* (CS-M), substituted for homologous pairs of *Gossypium hirsutum* (TM-1) chromosome or chromosome arm. The eleven CS lines utilized for this study were: CS-B26Lo, CS-T17, CS-B16-15, CS-B17-11, CS-B12, CS-T05sh, CS-T26Lo, CS-T11sh, CS-M11sh, CS-B22sh, and CS-B22Lo. The parent line TM-1 and two commonly grown cotton (Enlist^®^ and UA48) were also included. The eleven CS lines, TM-1, Enlist^®^, and UA48, are considered donor species screened for their weed suppressive abilities pertaining to Palmer amaranth, which was the recipient species. Palmer amaranth seeds were acquired from Aslan Seed Company and assumed to be a susceptible population.

### 4.2. Germination and Preparation of Plants Used in the Stairstep Structure

All plants in the study (CS lines, control varieties, Palmer amaranth) were germinated in a growth chamber using rockwool. The rockwool was soaked for 30 min n water adjusted to have a pH of 5.0. The seeds were then inserted into the rockwool pieces and placed in a dixie tray covered with a greenhouse top to maintain moisture levels. The growth chamber was set at 53% humidity, with a day-night cycle of 16/8.

**Figure 3:**
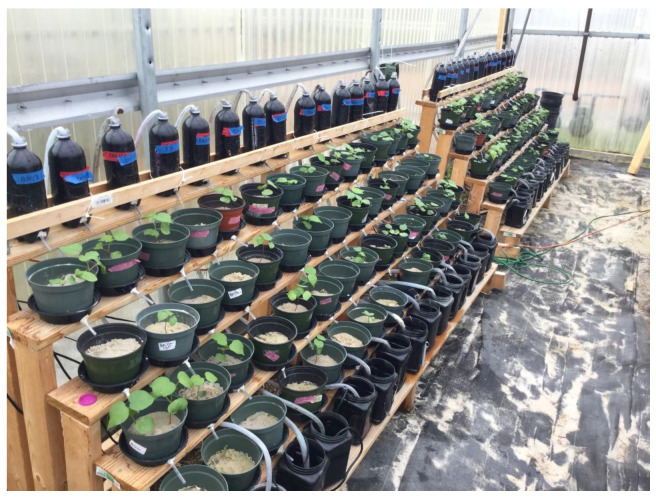
A photograph of the stair-step structure utilized in this experiment for screening allelopathy in cotton. The structure is composed of six platforms. The first platform is outfitted with bottles and tubes. The second, third, fourth, and fifth platforms hold the potted plants used in the experiment. The sixth and final platform supports the collection tank. Each column is outfitted with a bottle, four pots, a collection tank, a water pump, and tubing connection the water pump on the sixth platform to the bottle on the first platform. The pump is set on a timer. Each column can be considered a closed-loop system and is independent of neighboring columns.

Upon germination, the seedlings were transplanted into pots of Quickrete Play Sand (silicon dioxide). Each pot was planted with three seedlings, and each pot was considered an experimental unit (which will be interchanged with the term ‘pot’ throughout this paper). The seedlings were given 2–3 weeks to establish in the sand. Once established, all experimental units were placed into a stair-step structure, as outlined by Schumaker et al. (2020) (Figure 3).

### 4.3. Design of Stairstep Structure

The stair-step construct consists of six descending platforms to place potted plants of interest. The first platform was outfitted with bottles. The second, third, fourth, and fifth platforms held the potted plants in the experiment (Figure 1). The sixth and final platform was equipped with a collection tank and a pump. The pump was set on a timer, and every 6 h, water was pumped through plastic tubing from the collection tank on the final platform to the corresponding bottle on the first platform. The water drained from the bottle into the descending potted plants until it drained into the collection tank. This cycle continued for the duration of the experiment. Every column was outfitted with its pump, tubes, and so forth so each column can be considered a closed-loop system and independent from one another. All water used in the experiment was distilled.

**Figure 4:**
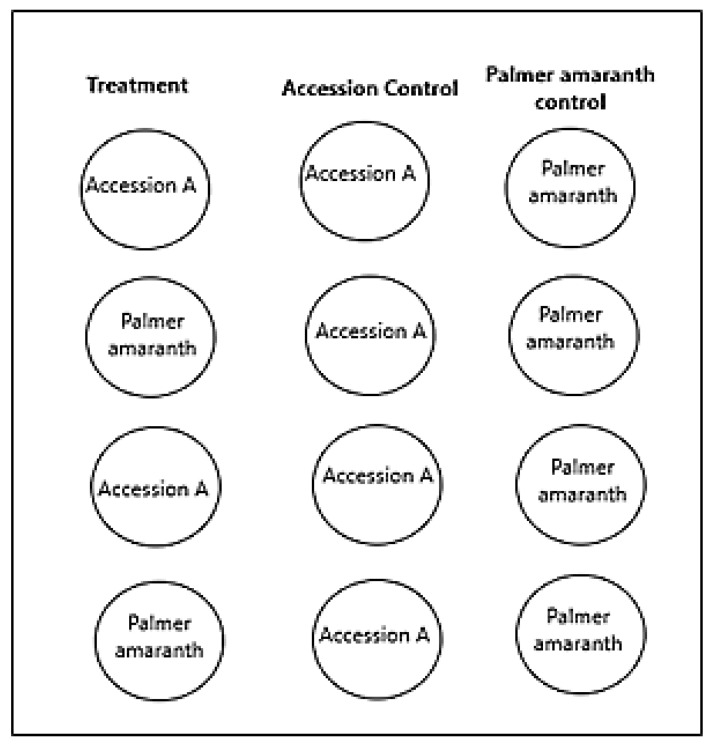
Diagrammatic representation of the stair-step setup. There are two columns for every CS line tested: the treatment column and the control column. The treatment column comprises four pots, two containing the same CS lines and two containing Palmer amaranth (PA). The treatment column starts with a pot of CS cotton, followed by a pot of PA, a pot of the same CS line, and a pot of PA. The control column is composed of four descending pots of the same CS line. This design was repeated for every CS line tested.

For every CS line tested, there were two columns: the treatment column and the control column (Figure 4). The treatment column was composed of four pots, two containing the same CS lines and two containing Palmer amaranth. The treatment column was alternated and started with a pot of CS cotton, followed by a pot of Palmer amaranth, a pot of the same CS line, and a pot of Palmer amaranth. The control column was composed of four descending pots of the same CS line. This design was repeated for every CS line tested.

### 4.4. Methods

Experiments were conducted in 2020 and 2021 in two runs in the greenhouse at the R.R. Foil Plant Science Research Center, Mississippi State University. The temperature in the greenhouse was set at 28 °C during the day and 24 °C at night with a 16/8 h day/night cycle. The system was fertilized using Hoagland’s No. 2 basal salts (Caisson Laboratories, Smithfield, UT, USA). On the day of the establishment (DOE), a starting concentration of 1500 mL quarter strength Hoagland’s solution was used to fill each tank, respectively. This was repeated 14 days after establishment (DAE). The heights of all plants were measured in centimeters at 1, 7, 14, and 21 DAE. At 21 DAE, chlorophyll concentrations were measured and recorded using a CCM300 chlorophyll meter (Opti-Sciences, Hudson, NH, USA).

### 4.5. Calculations

Palmer amaranth height reduction was calculated using the following formula:

Height reduction (%) = [height of control PA (cm) − height of experimental PA (cm)/height of control PA (cm)] × 100

Palmer amaranth chlorophyll reduction was calculated using the following formula:

Chlorophyll concentration [cc] reduction (%) = [cc of control PA (unit) − cc of experimental PA (unit) /cc of control PA (unit)] × 100

where the height of the control Palmer amaranth is the mean value for all Palmer amaranth plants in the four control pot. The experimental Palmer amaranth values were derived from the mean parameter value of all PA plants present in an experimental column per CS line.

### 4.6. Statistical Analysis

The experimental units were arranged in a randomized complete design with three replications. The experiment was run two times. Accessions were determined to be the fixed effect, and replications were understood to be the random effect. Data for height reduction values at 14 DAE and chlorophyll reduction at 21 DAE were analyzed separately by employing a general linear model, and mean values were separated using Fisher’s Protected Least Significant Difference at or below a 0.05 probability level in JMP 14 (JMP^®^, Version 13. SAS Institute Inc., Cary, NC, USA, 1989–2007). Hierarchal clustering guided by K means clustering was applied to visualize correlation among and between variables and components in JMP. This technique clustered the CS lines into associations based on Palmer amaranth inhibition (height and chlorophyll concentration). The data were explored through principal component analyses.

## 5. Conclusions

Future studies should include a wider variety of parameters to screen cotton and Palmer amaranth interactions. It would be especially useful to periodically sample the water from each column in the stair-step structure to analyze using High-Performance Liquid Chromatography (HPLC). This would provide us with a means to identify chemicals present in the nutrient solution throughout the duration of the experiment. It would also provide greater insight into the inducible nature of allelopathic phenomena. Additional parameters to consider are dry biomass, root length, and seed production. The use of greenhouse lights would provide environmental control throughout the study’s duration. Furthermore, the system could be utilized to test cotton varieties against multiple weed species. Field experiments with the selected CS lines to suppress the weed growth and development will be helpful to confirm the preliminary results of this research.

Allelopathic cotton varieties could be a valuable tool for farmers battling herbicide-resistant weeds or otherwise seeking alternative weed control techniques [23]; however, allelopathic crops need to be competitive in a field setting, which would be most effective when the crop is mature enough to produce a canopy. In these situations, it would be advantageous to use chemical weed control during the critical weed-free period and rely on allelopathic chemicals later in the season [36]. The discovery of novel cotton germplasm with the genetic potential to impede weed growth and development will benefit the farmers on the frontlines in the battle against the evolution of herbicide resistance weedy species.

## Figures and Tables

**Table 1:** Palmer amaranth (PA) height and chlorophyll reduction values in the presence of cotton chromosome substitution (CS) lines.

Cotton Accession	PA Mean Height Reduction 7 DAE (%)	PA Mean Height Reduction 14 DAE (%)	PA Mean Height Reduction 21 DAE (%)	PA Mean Chlorophyll Reduction (%)	Mean Susceptibility (%)
CS-T05sh	22.94	32.92	**24.06 c**	9.78	22.43
CS-B12	12.17	41.50	**24.70 c**	19.66	24.51
CS-B26Lo	24.65	35.27	**28.24 c**	8.98	24.29
UA48	10.68	27.71	**28.24 c**	16.50	20.78
TM-1	21.66	44.96	**30.58 c**	11.85	27.26
CS-B16-15	20.82	46.23	**33.34 c**	9.81	27.55
CS-B22Lo	20.13	41.72	**33.42 c**	21.18	29.11
CS-T17	30.23	39.70	**36.77 c**	12.85	29.89
CS-B10	29.65	38.11	**36.92 c**	16.49	30.30
CS-M11sh	30.10	44.42	**37.15 c**	10.46	30.53
CS-T11sh	23.17	42.17	**38.60 c**	17.00	30.24
CS-B17-11	27.73	35.71	**40.42 bc**	10.17	28.51
Enlist^®^	19.18	44.60	**53.20 abc**	26.49	35.87
CS-T26Lo	21.93	71.52	**68.32 ab**	16.42	44.55
CS-B22sh	55.23	88.11	**76.82 a**	20.07	60.06

Mean height reduction (7, 14, and 21 DAE) and chlorophyll concentration reduction (21 DAE) of recipient Palmer amaranth species in the stair-step structure. The means were separated using Fisher’s Protected LSD (α = 0.05) with significant and linked groups marked in the table with corresponding letters. Columns in bold type font and coupled with letters are the only times in which differences were calculated to be statistically significant. The mean susceptibility is derived from the height reduction and chlorophyll concentration reduction of Palmer amaranth for each CS line tested.

**Table 2:** Mean cotton height and chlorophyll reduction values in the presence of Palmer amaranth.

Cotton Accession	Cotton Height Reduction 7 DAE (%)	Cotton Height Reduction 14 DAE (%)	Cotton Height Reduction 21 DAE (%)	Cotton Chlorophyll Reduction (%)	Mean Susceptibility (%)
CS-B22sh	4.99	**14.41 abc**	**2.56 c**	0.47	5.61
CS-B16-15	18.19	**6.54 c**	**9.28 bcd**	3.13	9.29
CS-T11sh	10.97	**5.43 c**	**3.58 cd**	2.87	5.71
CS-T26Lo	10.50	**26.93 ab**	**9.13 bcd**	15.02	15.40
CS-B17-11	26.29	**3.18 c**	**2.53 d**	3.50	8.88
CS-M11sh	16.91	**10.16 c**	**6.59 bcd**	5.92	9.90
CS-B10	21.57	**14.10 bc**	**6.55 bcd**	2.86	11.27
UA48	5.16	**12.25 bc**	**7.65 bcd**	0.41	6.37
TM-1	3.25	**12.25 bc**	**3.41 cd**	4.93	5.96
Enlist^®^	16.67	**4.66 c**	**21.61 ab**	5.27	12.05
CS-T17	12.61	**4.09 c**	**3.94 cd**	4.76	6.35
CS-T05sh	4.65	**5.84 c**	**5.49 cd**	3.33	4.83
CS-B26Lo	11.70	**9.79 c**	**16.51 abc**	2.40	10.10
CS-B22Lo	16.40	**31.96 a**	**31.33 a**	4.07	20.94
CS-B12	16.50	**5.58 c**	**9.08 bcd**	4.88	9.01

Mean height reduction (7, 14, and 21 DAE) and chlorophyll concentration reduction (21 DAE) of donor species *Gossypium hirsutum* in the stair-step structure. The means were separated using Fisher’s Protected LSD (α = 0.05) with significant and linked groups marked in the table with corresponding letters when appropriate. Columns in bold type font and coupled with letters are the only times in which differences were calculated to be statistically significant The mean susceptibility is derived from the height reduction and chlorophyll concentration reduction for each cotton line tested.

## References

[1] Zimdahl R.L. (2018). Fundamentals of Weed Science.

[2] Buchholtz K.P. (1967). Report of the terminology committee of the Weed Science Society of America. Weeds.

[3] Humburg N.E., Colby S.R. (1989). Herbicide Handbook of the Weed Science Society of America.

[4] Culpepper A.S., Webster T.M., Sosnoskie L.M., York A.C., Nandula V.K. (2010). Glyphosate-resistant Palmer amaranth in the United States. Glyphosate Resistance in Crops and Weeds: History, Development, and Management.

[5] Morgan G.D., Baumann P.A., Chandler J.M. (2001). Competitive Impact of Palmer Amaranth (*Amaranthus palmeri*) on Cotton (*Gossypium hirsutum*) Development and Yield. Weed Technol..

[6] Webster T.M., Nichols R.L. (2012). Changes in the prevalence of weed species in the major agronomic crops of the Southern United States: 1994/1995 to 2008/2009. Weed Sci..

[7] Braxton L.B., Richburg J.S., York A.C., Culpepper A.S., Haygood R.A., Lovelace M.L., Walton L.C. (2017). Resistance of Enlist™(AAD-12) Cotton to Glufosinate. Weed Technol..

[8] Smith D.T., Baker R.V., Stelle G.L. (2000). Palmer Amaranth (*Amaranthus palmeri*) Impacts o Yield, Harvesting, and Ginning in Dryland Cotton (*Gossypium hirsutum*). Weed Technol..

[9] Appleby A.P. (2005). A history of weed control in the United States and Canada—A sequel. Weed Sci..

[10] Webster T.M., Sosnoskie L.M. (2010). Loss of glyphosate efficacy: A changing weed spectrum in Georgia cotton. Weed Sci..

[11] Srinivasan P.R., Shigeura H.D.T., Sprecher M., Sprinson D.B., Davis B.D. (1956). The biosynthesis of shikimic acid from D-glucose. J. Biol. Chem..

[12] Xu J., Smith S., Smith G., Wang W., Li Y. (2019). Glyphosate contamination in grains and foods: An overview. Food Control..

[13] Székács A., Darvas B. (2018). Re-registration challenges of glyphosate in the European Union. Front. Environ. Sci..

[14] Mesnage R., Bernay B., Séralini G.E. (2013). Ethoxylated adjuvants of glyphosate-based herbicides are active principles of human cell toxicity. Toxicology.

[15] VanGessel M.J. (2001). Glyphosate-resistant horseweed from Delaware. Weed Sci..

[16] Comont D., Hicks H., Crook L., Hull R., Cocciantelli E., Hadfield J., Neve P. (2019). Evolutionary epidemiology predicts the emergence of glyphosate resistance in a major agricultural weed. New Phytol..

[17] Tranel P.J., Wright T.R., Heap I.M. (2019). The International Survey of Herbicide Resistant Weeds. www.weedscience.org/Summary/MOA.aspx?MOAID=3.

[18] Norsworthy J.K., Griffith G.M., Scott R.C., Smith K.L., Oliver L.R. (2008). Confirmation and control of glyphosate-resistant Palmer amaranth (*Amaranthus palmeri*) in Arkansas. Weed Technol..

[19] Norsworthy J.K., Ward S.M., Shaw D.R., Llewellyn R.S., Nichols R.L., Webster T.M., Barrett M. (2012). Reducing the risks of herbicide resistance: Best management practices and recommendations. Weed Sci..

[20] Charles G., Taylor I., Roberts G., Sindel B.M., Johnson S.B. (2004). The impact of the cotton farming system on weed succession: Implications for herbicide resistance and adoption of an integrated weed management approach. Proceedings of the 14th Australian Weeds Conference.

[21] Kong C.H., Chen X.H., Hu F., Zhang S.Z. (2011). Breeding of commercially acceptable allelopathic rice cultivars in China. Pest. Manag. Sci..

[22] Sun B., Kong C.H., Wang P., Qu R. (2012). Response and relation of allantoin production in different rice cultivars to competing barnyardgrass. Plant. Ecol..

[23] Rizvi S.J. (2012). Allelopathy: Basic and Applied Aspects.

[24] Hoffman M.L., Weston L.A., Snyder J.C., Regnier E.E. (1996). Separating the effects of sorghum (*Sorghum bicolor*) and rye (*Secale cereale*) root and shoot residues on weed development. Weed Sci..

[25] Gui-Ying J., Jian-Guo L., Yan-Bin L. (2015). Allelochemicals from cotton (*Gossypium hirsutum*) rhizosphere soil: Inhibitory effects on cotton seedlings. Allelopath. J..

[26] Hall A.B., Blum U., Fites R.C. (1982). Stress modification of allelopathy of *Helianthus annuus* L. debris on seed germination. Am. J. Bot..

[27] Saha S., Jenkins J.N., Wu J., McCarty J.C., Gutiérrez O.A., Percy R.G., Stelly D.M. (2006). Effects of chromosome-specific introgression in upland cotton on fiber and agronomic traits. Genetics.

[28] Chen Z.J., Scheffler B.E., Dennis E., Triplett B.A., Zhang T., Guo W., Paterson A.H. (2007). Toward sequencing cotton (*Gossypium*) genomes. Plant. Physiol..

[29] Wendel J.F., Brubaker C.L., Percival A.E. (1992). Genetic diversity in Gossypium hirsutum and the origin of upland cotton. Am. J. Bot..

[30] Saha S., Raska D.A., Stelly D.M. (2006). Upland Cotton (*Gossypium hirsutum* L.) x Hawaiian Cotton (*G. tomentosum* Nutt. Ex. Seem.) F_1_ hybrid hypoaneuploid chromosome substitution series. J. Cotton Sci..

[31] Saha S., Stelly D.M., Raska D.A., Wu J., Jenkins J.N., McCarty J.C., Campbell B.T. (2011). Chromosome substitution lines: Concept, development and utilization in the genetic improvement of Upland cotton. Plant. Breed. InTech Slavka Krautzeka.

[32] Wu Z., Soliman K.M., Bolton J.J., Saha S., Jenkins J.N. (2008). Identification of differentially expressed genes associated with cotton fiber development in a chromosomal substitution line (CS-CS-B22sh). Funct. Integr. Genom..

[33] Brubaker C.L., Bourland F.M., Wendel J.F. (1999). The origin and domestication of cotton. Cotton: Origin, History, Technology, and Production.

[34] Awasthi A., Reddy K.R., Saha S., Jenkins J.N., Stelly D.M. (2018). Morph-physiological responses of cotton interspecific chromosome substitution lines to low temperature and drought stresses. Euphytica.

[35] Weiner J. (2004). Allocation, plasticity and allometry in plants. Perspectives in Plant Ecology. Evol. Syst..

[36] Kruse M., Strandberg M., Strandberg B. (2000). Ecological Effects of Allelopathic Plants-A Review.

[37] Schumaker B.C., Stallworth S., de Castro E., Fuller M.G., Shrestha S., Tseng T.M. (2016). Repeatable Stair-Step Assay to Access the Allelopathic Potential of Weedy Rice (*Oryza sativa* ssp.). JoVE (J. Vis. Exp.).

[38] Camberato J.J. (2007). Cation exchange capacity-everything you want to know and much more. Magnesium.

[39] Bednarz C.W., Oosterhuis D.M., Evans R.D. (1998). Leaf photosynthesis and carbon isotope discrimination of cotton in response to potassium deficiency. Environ. Exp. Bot..

[40] Wu H., Pratley J., Lemerle D., Haig T. (2001). Allelopathy in wheat (*Triticum aestivum*). Ann. Appl. Biol..

[41] Putnam A.R., DeFrank J., Barnes J.P. (1983). Exploitation of allelopathy for weed control in annual and perennial cropping systems. J. Chem. Ecol..

